# Ceramide-Lowering Therapy Improves Metabolic Health in Aged UM-HET3 Mice Fed a Western Diet

**DOI:** 10.1093/geroni/igaf122.4086

**Published:** 2025-12-31

**Authors:** Hamid Koroni, Adolfo Garcia, Jiyuan Yin, Erik Marchant, Vivian Diaz, Blake Rasmussen, Shangang Zhao, Juan Palavicini

**Affiliations:** University of Texas Medical Branch at Galveston, League City, Texas, United States; University of Texas Health Science Center at San Antonio, San Antonio, Texas, United States; University of Texas Health Science Center at San Antonio, San Antonio, Texas, United States; University of Texas Health Science Center at San Antonio, San Antonio, Texas, United States; University of Texas Health Science Center at San Antonio, San Antonio, Texas, United States; University of Texas Health Science Center at San Antonio, San Antonio, Texas, United States; University of Texas Health Science Center at San Antonio, San Antonio, Texas, United States; University of Texas Health Science Center at San Antonio, San Antonio, Texas, United States

## Abstract

Aging is the leading risk factor for virtually all chronic diseases, and when combined with obesity from high-fat, high-carbohydrate Western diets, accelerates metabolic decline. Ceramide, lipids whose synthesis is regulated by the longevity-assurance-gene (LAG1), accumulate with age and in metabolic syndrome, impairing glucose and fatty acid metabolism and contributing to insulin resistance, mitochondrial dysfunction, inflammation, and cellular senescence. This study tested whether blocking ceramide synthesis could improve metabolic function in aged, genetically diverse UM-HET3 mice fed a Western diet. Aged mice received myriocin, a serine palmitoyl transferase inhibitor, intermittently (one week on, two weeks off) alongside a Western diet. Comparison groups included Western diet alone, a control diet without added sucrose/cholesterol but similar in calories, and standard chow. Metabolic activity was assessed with the Promethion Metabolic System, analyzing three full days of data. Myriocin increased fatty acid utilization compared to control diet and chow, and showed an increasing trend relative to Western diet, with no differences in energy expenditure. Oral Glucose tolerance tests (OGTT), blood lactate and ketones, indicated that myriocin restored glucose tolerance to control diet levels, without affecting lactate or ketones. Body composition by qMRI showed reduced body fat percentage with myriocin. Liver mitochondrial function assessed by high-resolution respirometry was negatively impacted by Western diet and partially rescued by myriocin. ELISA demonstrated normalized insulin levels without changes in leptin. By modeling the human-like Western diet in a genetically diverse animal model, these findings provide proof-of-concept that targeting ceramide synthesis can extend healthspan in aged, metabolically unhealthy mice.

